# Impact Protection Potential of Mammalian Hair: Testing the Pugilism Hypothesis for the Evolution of Human Facial Hair

**DOI:** 10.1093/iob/obaa005

**Published:** 2020-04-15

**Authors:** E A Beseris, S E Naleway, D R Carrier

**Affiliations:** 1 Department of Biology, University of Utah, 257 S. 1400 E, Salt Lake City, UT 84112, USA; 2 Department of Mechanical Engineering, University of Utah, 100 S. 1495 E, Salt Lake City, UT 84112, USA

## Abstract

Because facial hair is one of the most sexually dimorphic features of humans (*Homo sapiens*) and is often perceived as an indicator of masculinity and social dominance, human facial hair has been suggested to play a role in male contest competition. Some authors have proposed that the beard may function similar to the long hair of a lion’s mane, serving to protect vital areas like the throat and jaw from lethal attacks. This is consistent with the observation that the mandible, which is superficially covered by the beard, is one of the most commonly fractured facial bones in interpersonal violence. We hypothesized that beards protect the skin and bones of the face when human males fight by absorbing and dispersing the energy of a blunt impact. We tested this hypothesis by measuring impact force and energy absorbed by a fiber epoxy composite, which served as a bone analog, when it was covered with skin that had thick hair (referred to here as “furred”) versus skin with no hair (referred to here as “sheared” and “plucked”). We covered the epoxy composite with segments of skin dissected from domestic sheep (*Ovis aries*), and used a drop weight impact tester affixed with a load cell to collect force versus time data. Tissue samples were prepared in three conditions: furred (*n* = 20), plucked (*n* = 20), and sheared (*n* = 20). We found that fully furred samples were capable of absorbing more energy than plucked and sheared samples. For example, peak force was 16% greater and total energy absorbed was 37% greater in the furred compared to the plucked samples. These differences were due in part to a longer time frame of force delivery in the furred samples. These data support the hypothesis that human beards protect vulnerable regions of the facial skeleton from damaging strikes.

## Introduction

As is the case in other species of great apes, human males perpetrate the vast majority of violence and most of these acts of aggression are directed at other males (Adams 1983; Chagnon 1988; Daly and Wilson 1988; Keeley 1996; Wrangham and Peterson 1996; Walker 2001; Puts 2010; Ellis et al. 2013; Hill et al. 2016). When human males fight hand-to-hand, the face is usually the primary target (Carrier and Morgan 2015). Consequently, it is not surprising that human males suffer substantially more injuries to the face from interpersonal violence than do females. Epidemiology studies of interpersonal violence indicate that males suffer 68–92% more injuries to the face from fights than do females (Shepherd et al. 1990; Bostrom 1997; Brink et al. 1998; Simsek et al. 2007; Czerwinski et al. 2008; Lee 2009; Allareddy et al. 2011; Suh and Kim 2012).

Because sexual dimorphism is often greatest in those phenotypes that enhance a male’s capacity to dominate other males (Clutton-Brock and Harvey 2009; Parker 1983; Andersson 1994), it is not surprising that the facial bones which suffer the highest rates of fracture from interpersonal violence are the parts of the skull that exhibit the greatest sexual dimorphism in both modern humans and early hominins (i.e., bipedal apes; Carrier and Morgan 2015). From the perspective of sexual selection, it is reasonable to suspect that these dimorphic facial features emerged as a result of male–male contest competition, and act to protect the face against damaging strikes (Puts 2010; Stirrat et al. 2012; Carrier and Morgan 2015; Puts et al. 2015; Puts 2016). Consistent with this suggestion is the observations that masculine facial structure is correlated with greater upper body strength (Fink et al. 2007; Sell et al. 2009; Windhager et al. 2011), aggressive behavior (Carré and McCormick 2008; Carré et al. 2009, 2010; Třebický et al. 2013), social dominance (Muller and Mazur 1997; Geniole et al. 2015), and reproductive success (Mazur et al. 1994).

Another trait that exhibits pronounced sexual dimorphism in humans is facial hair (Darwin 1871; Dixson et al. 2018). Among our closest relatives, the African apes (chimps, bonobos, and gorillas), facial hair is equally prominent in males and females (Darwin 1871). Relative to the African apes, human females have significantly reduced facial hair, whereas at puberty human males develop continuously growing hair that covers the front of the upper jaw (mustache) and the anterior neck and lower jaw (beard; Darwin 1871; Dixson et al. 2005; Dixson et al. 2017). As is true for masculine skeletal features, men with full beards are reportedly perceived as being more masculine, socially dominant, and behaviorally aggressive in comparison to clean-shaven men (Neave and Shields 2008; Dixson and Vasey 2012; Dixson and Brooks 2013; Saxton et al. 2016; Sherlock et al. 2017; Třebický et al. 2019). In addition, human male facial hair has been shown to positively impact mating success in highly competitive environments (Barber 2001; Dixson et al., 2017).

Some authors suggest these relationships are due to facial hair enhancing the size and appearance of the sexually dimorphic regions of the face—most notably the mandible and maxilla (Guthrie 1970; Muscarella and Cunningham 1996; Neave and Shields 2008; Dixson et al. 2017, 2018). Others have proposed that the beard actually serves to protect the throat and jaw during fighting (Blanchard 2010). In this context, the mane of male lions offers an intriguing possible analogy. Like human beards, lion manes are specific to males. The very thick hair of a lion’s mane could provide protection from an attacker’s teeth or it might make the head, neck, and chest more difficult for an attacker to grab and hold with the claws of his forelimbs so that it could deliver a damaging bite with his jaws. Indeed, Darwin (1871) suggested that manes of male lions, Canadian lynx, baboons, sea lions, bison, and elk provide physical protection in male–male fights. (In contrast, when considering humans, Darwin speculated that the beard evolved as an “ornament” favored by females.) More recently, Blanchard (2010) has argued that the manes of lions may “mitigate” the danger of fights among pride males by making the existence of multi-male and female groups possible facilitat protection of prides against take-overs and infanticide by nomadic males. In contrast, West et al. (2006) compared patterns of injury, mane development, and adult mane morphology in African lions and found no evidence that the mane conferred effective protection against wounding. However, they also argue that their results suggest that “the general mane area is not a target, but hint that attackers avoid the mane, or that the mane protects this area from attack.” Thus, the extent to which the mane of lions is protective remains unresolved.

The suggestion that human beards may provide protection in a fight is consistent with the observations that (1) the mandible, which is superficially covered by the beard, is one of the most commonly fractured facial bones in interpersonal violence (Shepherd et al. 1990; Bostrom 1997; Lee 2009; Hojjat et al. 2016) and (2) a fractured mandible, prior to modern surgical methods, likely represented a relatively grave facial injury. Based on these observations, we hypothesized that human facial hair provides physical protection from strikes that would cause blunt trauma. Specifically, we predicted that thick facial hair reduces the amount of force that underlying tissues experience from a strike due to absorption and dispersal of energy of the strike.

## Methods

Human bone tissue was modeled using a short fiber epoxy composite bone analog (manufactured by Pacific Research Laboratories, Inc., Vashon, WA), which has material properties similar to human cortical bone (Cuppone et al. 2004; Chong et al. 2007). Because it was not practical to obtain fully bearded skin samples from human cadavers, and loose human hair was anticipated to not distribute the force of impact the way *in situ* hair may, we used skin samples from domestic sheep (*Ovis aries*) purchased from a local slaughterhouse. Sheep fleece is not a perfect analogy for the hair of human beards. The follicles of sheep fleece average one fourth the diameter of human beard hair (∼18 μm versus 75 μm; Bosman 1934; Floyd et al. 2018) and are much more densely packed (6000 follicles per cm^2^ versus 70 follicles per cm^2^; Bosman 1934; Maurer et al. 2016). This represents a five-fold greater cross-sectional area of hair follicles for fleece than beards. However, the follicles of full human beards are often more than five-fold longer than the follicles of the sheep fleece samples that we tested (3.30 ± 1.04 cm, mean and standard deviation [SD]). Consequently, the volume of follicles in our fleece samples did approximate the volume of full beards which is unlikely to be true for the pelts of most other species.

The bone analog was cut into small rectangles with dimensions 60 mm × 65 mm × 3 mm and covered by sheepskin. Skin samples were cut to the same dimensions as the fiberglass and were soaked in a saline solution (0.9% NaCl) for at least one hour prior to testing to ensure the skin had the same water content as living tissue. Hydration level has been shown to have significant effects on the material properties of organic matter, and therefore must be standardized for all samples (Lee et al. 2011; Trim et al. 2011). Care was taken to keep the hair of the samples dry. The hair of the sheepskin samples was prepared in three separate conditions: sheared, plucked, and furred. Sheared samples were trimmed with manual sheep shears to ∼0.5 cm in length. Sheared samples were included to test whether the presence of hair roots in the skin influenced the results. Plucked samples had all hair fibers removed, including the roots. Furred samples were not manipulated in any way, and had an approximate hair length of 8 cm. Of note, these three conditions result in different total volumes and masses of hair and were chosen to best represent states that would occur in human males (i.e., full beard, trimmed beard, and hairless).

**Fig. 1:**
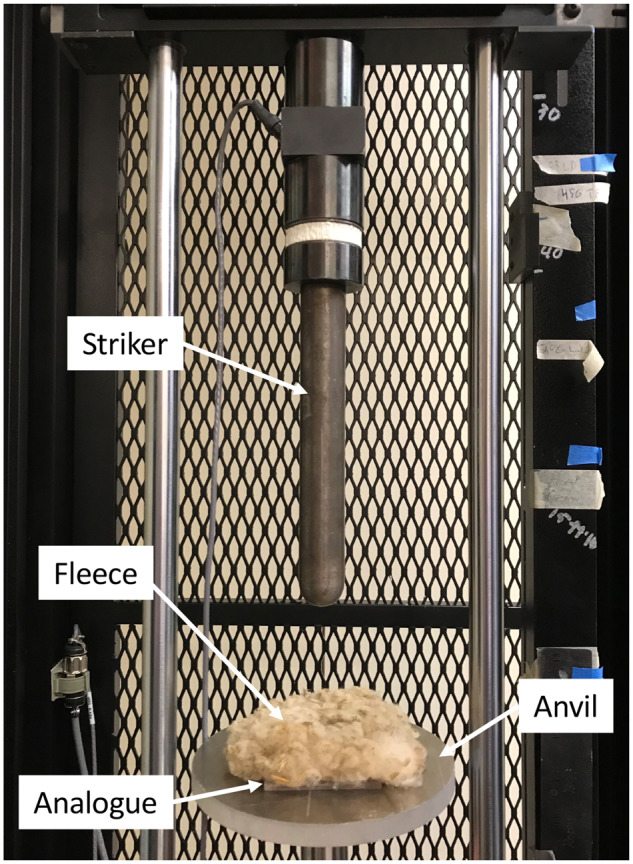
Photograph of the experimental setup using an Instron Dynatup 8250 drop weight impact tester.

All data were obtained by using a drop-weight impact test on an Instron Dynatup 8250 drop weight impact tester (Instron Corporation, Norwood, MA; Fig. 1). All tests were performed in accordance with ASTM Standard D5420 (ASTM Standard D5420-16 2016). This test involves dropping a blunt striker (diameter ∼3 cm, mass = 4.70 kg), from a known height toward a material sample mounted on an anvil. The anvil had a 55 mm × 50 mm hole to allow free suspension of the sample and to avoid effects of the contact between the anvil and sample that could alter the results. The Instron Dynatup Impulse data acquisition system (Instron Corporation, Norwood, MA) takes measurements from a 200 kN load cell to generate a graph of load (kN) versus time (ms). A velocity detector was also used to measure the instantaneous velocity (m/s) of the striker head at the time of impact.

Prior to obtaining data to compare across the three conditions, a standard drop height was determined. Starting from 5 cm above the anvil, the striker head was dropped onto a furred sample. If the sample showed signs of failure, the striker head was lowered an additional centimeter. If the sample did not show signs of failure, the striker head was raised an additional centimeter. Failure was defined as the point at which the fiberglass sample shows any cracks, fractures, holes, or dislodged shards. The striker head mass was not changed during the entire duration of testing. This process was repeated for 20 samples, following the approach of the ASTM Standard D5420 (ASTM Standard D5420-16 2016). From these data, the mean failure height was calculated by using Equation (1):

$h=h_{0}+d_{h}(\frac{A}{N}\pm 0.5)$, where

h= mean failure height (mm)

$d_{h}=$ increment of height (mm)

N= total number of failures or non-failures, whichever is smaller

$h_{0}=$ lowest height at which failure or non-failure occurred (mm)

$A=\sum_{i=0}^{k}in_{i}$, where i= integer. $n_{i}=$ number of events occurring at $h_{i}$, and $h_{i}=h_{0}+id_{h}$

Using this approach, the mean failure height was determined to be 7.4 cm (Supplementary Table S1), and the drop tower height was set to this height for the entire series of experimental tests. Twenty samples for each condition (shaved, plucked, and furred) were tested. Using the resultant load data (kN) and the mass of the striker (4.7 kg), the acceleration of the striker head (m/s^2^) was determined using Newton’s Second Law (*F* = *m* × *a*). The resultant acceleration dataset was integrated across the impact time frame to yield the instantaneous velocity (m/s) for each time frame, and subsequently, the kinetic energy (J). The energy absorbed by the sample (J) was calculated from the amount of kinetic energy lost by the striker head from the start of impact to the end of impact. From these data, the peak force in Newtons (PF), peak energy in Joules (PE), time to peak force in milliseconds (TPF), and time to peak energy in milliseconds (TPE) were recorded for each test.

**Table 1:** Frequency of failure for each condition

	Frequency of failure
Furred	0.45
Plucked	1
Sheared	0.95

A series of two-sample, single-tailed, unequal variance *t*-tests were used to determine statistical significance between raw PF, PE, TPF, and TPE data. We assumed the results were significantly different when the *P*-value was <0.05. Percent difference was also calculated for each of the four metrics between conditions, along with mean and SD for each condition. All data calculations, statistical analyses, and graphs were performed using Microsoft Excel (Microsoft Corporation, Redmond, WA).

### Statement on human and animal rights 

This research did not involve human or animal subjects.

## Results

The furred samples provided greater protection against impact than did the plucked or sheared samples (Table 1). Under the condition of the study in which the loading was set so that ∼50% of the furred samples would fail on impact, all of the plucked samples, 95% of the sheared samples, and 45% of the furred samples failed.

**Fig. 2:**
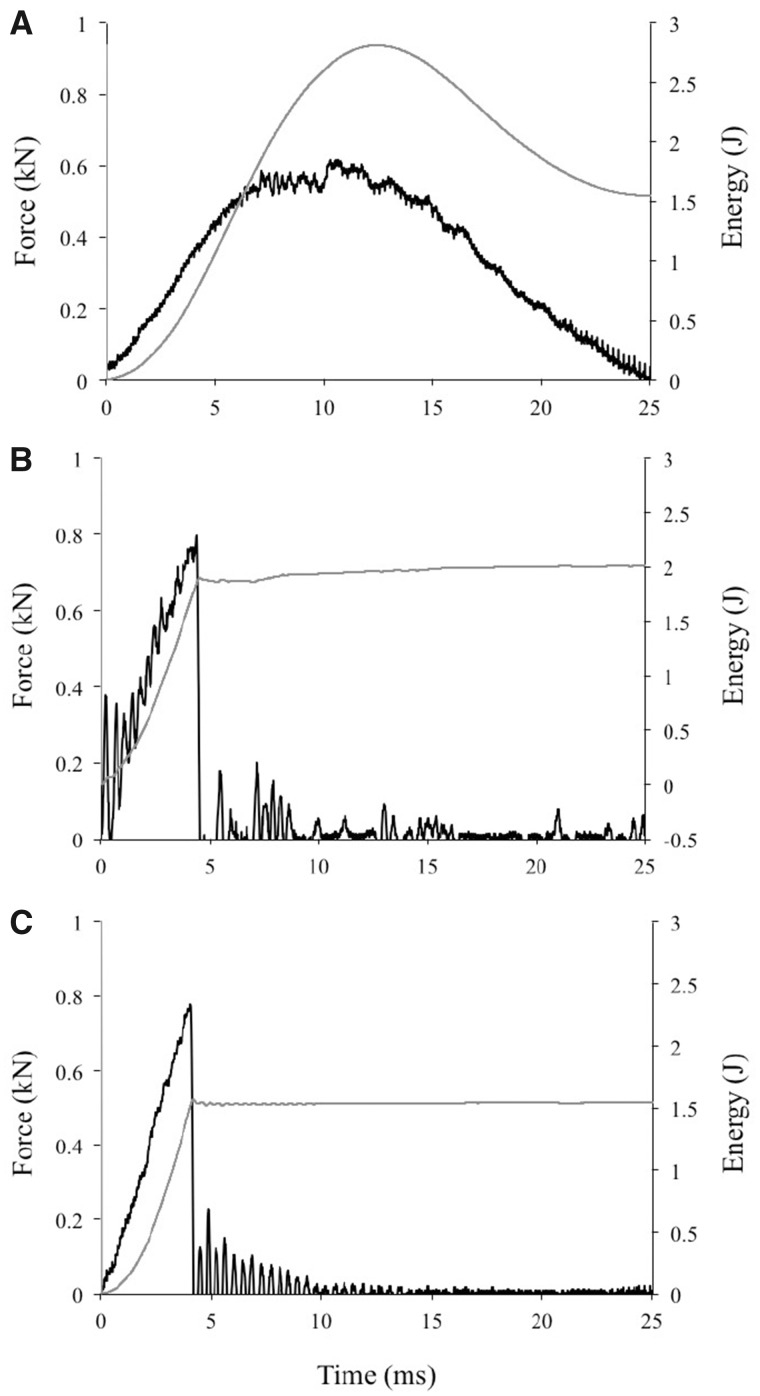
Representative graphs of impact force (black line) and energy (gray line) versus time for (**A**) a furred sample, (**B**) a sheared sample, and (**C**) a plucked sample.

**Fig. 3:**
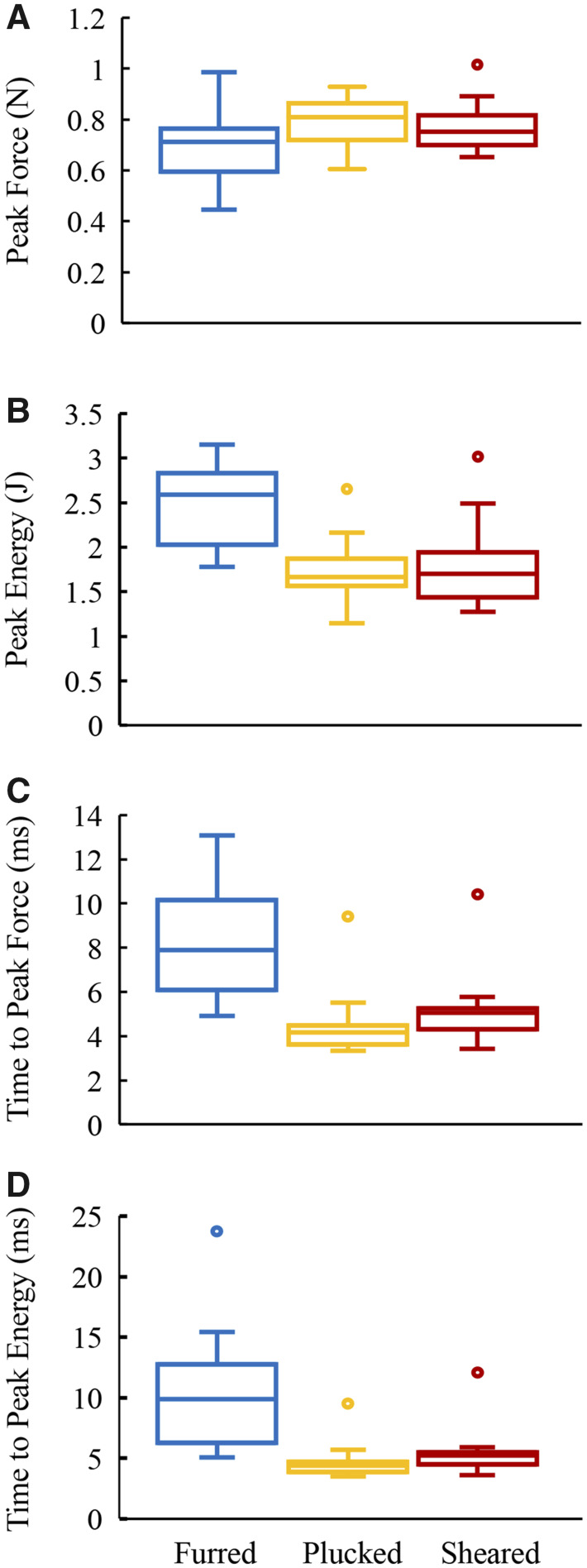
Box and whisker plots showing median, first and third quartiles, and minimum and maximum values of (**A**) peak force (kN), (**B**) peak energy (J), (**C**) time to peak force (ms), and (**D**) time to peak energy (ms) for each of the three conditions.

Example recordings of force and energy absorbed for impact tests of the furred, plucked, and sheared skin samples are shown in Fig. 2. As can be seen in these traces, the average peak force was significantly lower, energy absorbed was higher, and the time to peak force and peak energy absorbed was substantially greater in the furred samples than in the sheared and plucked samples (Fig. 3 and Table 2).

**Table 2:** Mean, SD, percent difference, and *P*-values for furred (F), plucked (P), and sheared (S) conditions

	Furred mean±SD	Plucked mean±SD	Sheared mean±SD	F × P %diff. (*P*)	F × S %diff. (*P*)	P × S %diff. (*P*)
PF (kN)	0.68 ± 0.16	0.79 ± 0.10	0.77 ± 0.09	15.60 (0.004)	12.79 (0.014)	2.82 (0.23)
PE (J)	2.46 ± 0.43	1.70 ± 0.34	1.80 ± 0.43	36.77 (<0.001)	31.24 (<0.001)	5.69 (0.211)
TPF (ms)	8.24 ± 2.40	4.38 ± 1.29	5.10 ± 1.40	61.17 (<0.001)	47.10 (<0.001)	15.16 (0.049)
TPE (ms)	10.30 ± 4.54	4.57 ± 1.28	5.36 ± 1.70	77.04 (<0.001)	63.14 (<0.001)	15.83 (0.054)

The greatest differences between the furred and plucked or sheared samples were observed in times to reach peak force and peak energy absorption (Fig. 3 and Table 2). The sheared and plucked samples were loaded more rapidly by impact and more often than not experienced loads that exceeded their breaking strength. This suggests that the greatest advantage offered by the hair is that it distributes the force of impact over a longer time frame.

The higher variation observed in the furred samples is largely due to differences in the samples that failed versus those that did not. Samples that did not fail (see Fig. 2) had PF, PE, TPF, and TPE values similar to the mean values for the furred condition (PF = 0.67 kN, PE = 2.46 J, TPF = 8.24 ms, TPE = 10.29 ms). In contrast, samples that did fail had much higher PF values (0.82 kN) and lower PE (1.91 J), TPF (5.26 ms), and TPE (5.39 ms) in comparison to the mean. The furred condition had a nearly equal amount of failures and non-failures (frequency of failure = 0.45), whereas the plucked and sheared conditions had nearly all failures (frequency of failure = 1 and 0.95, respectively). 

## Discussion

Our results show that on average the furred samples absorbed nearly 30% more energy than the sheared and plucked samples. Furred samples experienced lower peak impact forces and were loaded more slowly. These factors contributed to a reduced rate of furred sample failure as compared to sheared and plucked samples. Thus, the results of this study indicate that hair is indeed capable of significantly reducing the force of impact from a blunt strike and absorbing energy, thereby reducing the incidence of failure. If the same is true for human facial hair, then having a full beard may help protect vulnerable regions of the facial skeleton from damaging strikes, such as the jaw. Presumably, full beards also reduce injury, laceration, and contusion, to the skin and muscle of the face. Although not tested in this study, it is also likely that the hair of beards helps deflect an oblique blow by reducing friction between the face and the object striking it. These protective functions of beards may provide an advantage in male contest competition, and therefore be selectively favored. This may also explain why facial hair is associated with high masculinity, social dominance, and behavioral aggressiveness, as it may function as a true indicator of level of invulnerablity to facial injury (Neave and Shields 2008; Dixson and Vasey 2012; Dixson and Brooks 2013; Saxton et al. 2016; Sherlock et al. 2017).

No measures were significantly different between the plucked versus sheared conditions, except for TPE (*P* = 0.049). We anticipated this result as the presence or absence of hair roots in the skin was expected to have little influence on impact protection.

Among the significant differences between sample conditions, the time to peak force and time to peak energy are likely the most salient. Furred samples absorbed the impact more slowly than the sheared and plucked samples. We suspect that this is a result of individual hair fibers taking up part of the load as the striker head descended toward the skin, slowing the striker head as it passed by. By loading the hair fibers in addition to the skin and bone, the force of impact may also be distributed over a larger surface area. This is a similar mechanism to how a Kevlar fiber vest distributes the force of an incoming bullet (Cheeseman and Bogetti 2003). Regardless, absorption of energy by the fur must explain why furred samples were able to absorb 37% more energy than sheared and plucked ones.

Our results appear to conflict with a recent study that demonstrated beards do not provide a performance advantage in mixed martial arts (MMA) fights as measured by number wins by knock-out and decision (Dixson et al. 2018). This carefully controlled and compelling study, compared rates of winning in 600 fights involving 395 fighters, found no evidence of a performance advantage provided by facial hair, and concluded that “beards represent dishonest signals of formidability that may serve to curtail the escalation of intra-sexual conﬂict through intimidation rather than providing advantages in direct combat.” It is sensible to test the protective effect of beards in MMA fighters because epidemiology indicates that the most common injuries in MMA fights are facial lacerations, fractures, and concussions (Lystad et al. 2014; Jensen et al. 2017). Although this is not the result we would have predicted based on our observation that thick hair reduces peak impact force and energy applied to the structure beneath the hair, the metric used in their study “number of wins by knockout or technical knockout” is not a direct measure of the rate of those injuries that may be reduced by full beards. Our results provide no evidence that beards provide protection against being knockedout, rather our results are presumed to be most relevant to skin lacerations and facial bone fractures. Finally, as Dixon and collaborators note, their finding that beards do not provide a performance advantage may be more relevant to professional fighters than non-professionals.

While our data are consistent with the hypothesis that hair can protect bone and skin from the damaging effects of a blunt strike, it should be noted that this may not be true in every case. Human facial hair has great variation across populations—individuals from Middle Eastern and Northern European ancestry are capable of growing thick, bushy beards, whereas people of East Asian and American Indian heritage have relatively little facial hair. The sheepskin used in our study was extremely thick and wooly, and is probably only a good model for a very full and long human beard. To our knowledge, no quantitative data exist on how coarseness, density, and thickness of human facial hair varies across populations. Future research should incorporate these measures to determine which types of facial hair may provide the best protection against impact.

It is unknown why human populations vary in their developed facial hair. In groups in which thick facial hair is not present in males, other selective forces may have acted against facial hair. These groups may have lower rates of contest competition between males, thereby negating the advantage of a beard or they may need to maximize bare skin surface area for efficient thermoregulation in hot environments. The fact that facial hair is sexually dimorphic in humans, with females lacking beards and mustaches, strongly suggests that there are real disadvantages to having thick facial hair. If there were no tangible disadvantages, selection for facial hair in males would have resulted in beards in both sexes (Lande 1980).

Additional studies are needed to ascertain the mechanism by which hair dampens the effects of impact. We theorized that the hair fibers absorbed energy from the impactor head as it passed by and by spreading the force over a larger area. This is supported by the furred samples having a longer average time to reach peak force and absorbing more energy. This could be further substantiated by using highspeed video to see exactly what the hair fibers are doing upon impact. This could also be accomplished by creating a model of the hair fibers and running simulations.

The results of this study are consistent with the suggestion that the sexually dimorphic facial hair of humans may have evolved in response to selection on male–male fighting performance. Similarly, although not tested here, our results also support the suggestion that the mane of male lion’s provides some level of protection from injury when males fight (Darwin 1871; West et al. 2006; Blanchard 2010) due to the capacity of hair to slow and expand the area of energy transfer. As mentioned in the Introduction, male beards are one of the most sexually dimorphic features of human anatomy (Darwin 1871; Dixson et al. 2018). Men with full beards are perceived as being more masculine, socially dominant, and behaviorally aggressive in comparison to clean-shaven men (Neave and Shields 2008; Dixson and Vasey 2012; Dixson and Brooks 2013; Saxton et al. 2016; Sherlock et al. 2017; Třebický et al. 2019). Additionally, facial hair has been shown to positively impact mating success in highly competitive environments (Barber 2001; Dixson et al. 2017). These observations are all consistent with the hypothesis that beards evolved to enhance fighting performance by providing protection to vulnerable aspects of the face. Indeed, aspects of the anatomy of the human facial skeleton, and sexual dimorphism in facial shape, have been suggested to have evolved as a result of male–male contest competition, and act to protect the face against damaging strikes (Puts 2010; Stirrat et al. 2012; Carrier and Morgan 2015; Puts et al. 2015).

More broadly, the results of this study add to a growing body of evidence suggesting that specialization for male fighting has played a significant role in the evolution of the musculoskeletal system of humans. For example, the short limbs (Carrier 2007), plantigrade foot posture (Carrier and Cunningham 2017), and bipedal posture of our earliest hominins ancestors (Carrier 2011), and the force–velocity tuning (Carrier et al. 2011) and size (Carrier et al. 2015) of the muscles of the human leg may also be associated with improved fighting performance. Of direct relevance to this study is the suggestion that the proportions of the human hand (Morgan and Carrier 2013; Horns et al. 2014), and human sexual dimorphism in both strength of the muscles of the arm (Morris et al. 2020) and facial shape (Carrier and Morgan 2015) are, at some level, a product of selection on performance during fighting with fists. Many of these anatomical traits distinguish hominins from the other great apes and all of them are associated with performance improvements in other non-fighting behaviors. Nevertheless, the fact that the appearance of hominins in the fossil record coincides with the appearance of a suite of anatomical traits that have been demonstrated to improve performance in behaviors important to human fighting suggests that specialization for physical aggression may have played an early and persistent role in the evolution of our lineage.

## Funding

This work was funded by National Science Foundation grant IOS-0817782 (to D.R.C.).

## Supplementary data

Supplementary data available at *IOB* online.

## Data archiving

Data will be archived on Dryad once the manuscript is accepted for publication.

## Supplementary Material

obaa005_Supplementary_Data
